# Artificial Intelligence in Anaesthesiology: Current Applications, Challenges, and Future Directions

**DOI:** 10.4274/TJAR.2025.252320

**Published:** 2025-12-22

**Authors:** Burhan Dost, Engin İhsan Turan, Muhammed Enes Aydın, Ali Ahıskalıoğlu, Madan Narayanan, Resul Yılmaz, Alessandro De Cassai

**Affiliations:** 1 Ondokuz Mayıs University Faculty of Medicine, Department of Anaesthesiology and Reanimation, Samsun, Türkiye; 2 University of Health Sciences Türkiye, Kanuni Sultan Süleyman Training and Research Hospital, Clinic of Anaesthesiology and Reanimation, İstanbul, Türkiye; 3 Atatürk University Faculty of Medicine, Department of Anaesthesiology and Reanimation, Erzurum, Türkiye; 4 Frimley Park Hospital, NHS Frimley Health Foundation Trust, Camberley, United Kingdom; 5 Necmettin Erbakan University Faculty of Medicine, Department of Anaesthesiology and Reanimation, Konya, Türkiye; 6 University of Padua (DIMED), Department of Medicine, Padua, Italy; 7 University Hospital of Padua, Institute of Anaesthesia and Intensive Care Unit, Padua, Italy

**Keywords:** Airway management, artificial intelligence, intensive care, neuroanaesthesia, perioperative care, pharmacology, regional anaesthesia

## Abstract

Artificial intelligence (AI) is rapidly transforming anaesthesiology through advances in machine learning, deep learning, and large language models. AI-driven tools now contribute to nearly every phase of perioperative care, including preoperative risk stratification, intraoperative monitoring, imaging interpretation, airway assessment, regional anaesthesia, and critical care. Applications such as automated American Society of Anesthesiologists classification, prediction of postoperative complications and intensive care unit needs, electroencephalography-based depth-of-anaesthesia estimation, and proactive haemodynamic management are reshaping clinical decision-making. AI-augmented echocardiography enhances chamber recognition and functional measurements, whereas computer vision systems support airway evaluation and ultrasound-guided regional anaesthesia by providing real-time anatomical identification and facilitating training. In critical care, AI models facilitate the early detection of sepsis, organ dysfunction, and haemodynamic instability, while improving workflow efficiency and resource allocation. AI is increasingly used in academic writing, data processing, and medical education, offering opportunities for personalised learning and simulation but raising concerns about accuracy and hallucinations. In this review, we aimed to summarise the current applications of AI in anaesthesiology, highlight the methodological, ethical, and practical challenges that limit its integration, and discuss future directions for its safe and effective adoption in perioperative care.

**Main Points**

• Artificial intelligence (AI) is increasingly being integrated into all phases of perioperative care, including preoperative assessment, monitoring, airway management, regional anaesthesia, and critical care.

• AI-driven tools can enhance prediction of perioperative risks, estimation of anaesthetic depth, optimisation of haemodynamics, and interpretation of imaging, potentially improving safety and precision.

• AI is becoming widely used in academic writing, education, and clinical decision support, but requires careful oversight to avoid inaccuracies and hallucinated content.

• AI will not replace anaesthesiologists, but is expected to augment clinical judgment and shift the specialty toward a more cognitive, supervisory, and data-informed practice.

• Future progress requires multicentre validation, transparent reporting, robust regulations, and structured AI training for clinicians to ensure safe, ethical, and effective integration.

## Introduction

Artificial intelligence (AI) is a branch of computer science dedicated to developing software and hardware capable of simulating human cognitive functions, such as problem solving, object recognition, reasoning, and decision-making.^1^ The term AI was first introduced by John McCarthy in 1956, who defined it as “the science and engineering of making intelligent machines.”^2^ In 2024, the Nobel Prize in Physics was awarded to John J. Hopfield and Geoffrey E. Hinton for their pioneering work on artificial neural networks, which underpin modern clinical decision support systems, patient-monitoring algorithms, and data-driven anaesthesia technologies.^3^ In the same year, the Nobel Prize in Chemistry recognised the transformative impact of AI on computational protein structure predictions. DeepMind’s AI-based system, AlphaFold, which accurately predicts the three-dimensional (3D) structure of proteins, has opened new frontiers in biomedical research. This innovation has remarkable potential for anaesthesiology, including identifying novel drug targets (e.g., G-protein-coupled receptors and ion channels), modelling anaesthetic-receptor interactions, accelerating drug discovery, and predicting toxicity or adverse effects.^4^

To understand how these systems operate across diverse domains, it is essential to outline the fundamental concepts that underpin modern AI. Machine learning (ML) refers to systems that learn patterns and relationships from data without being explicitly programmed. Deep learning (DL), a subfield of ML, uses multi-layered artificial neural networks to enable hierarchical feature extraction and complex pattern recognition in data. Natural language processing (NLP) focuses on enabling machines to understand and generate human language. Depending on the learning paradigm, AI models can be trained in different ways: in supervised learning, they learn from labelled data with known outcomes; in unsupervised learning, they identify hidden structures or clusters without prior labels; in reinforcement learning, they improve performance through trial and error by maximizing a defined reward signal.^5^

Since its conceptual origins, AI has evolved across multiple domains; however, the introduction of transformer-based architectures in 2018 (e.g., GPT-1) marked a major turning point in this field. Building on these foundations, the advent of multimodal large language models (LLMs) in 2025 enabled AI systems to process and integrate diverse data modalities, including text, medical images, audio signals, and video recordings, thereby opening a new era of multimodal intelligence. In medicine, such models support imaging interpretation, drug discovery, diagnostic reasoning, treatment optimisation, and even surgical automation.^6^ Within anaesthesiology, AI applications are rapidly expanding across the perioperative continuum, prompting the question of whether intelligent systems may complement or even replace human anaesthesiologists.

In this review, we aimed to summarise the current applications of AI in anaesthesiology, to discuss its methodological and ethical challenges, and to explore future directions for its integration into perioperative care.

### Preoperative Assessment

The preoperative period represents a critical opportunity to enhance patient safety, anticipate perioperative risks, and optimise healthcare resources. By synthesising data on patient demographics, comorbidities, laboratory findings, and surgical variables, AI-driven systems can assist clinicians in identifying high-risk patients and predicting postoperative complications earlier and more accurately than conventional methods. This growing integration positions AI not only as a supportive analytical tool but also as a potential partner in clinical reasoning, capable of standardising decision-making in a field where subjective variability has long influenced outcomes.

One of the most fundamental risk classification systems used preoperatively is the American Society of Anesthesiologists (ASA) physical status classification. The ASA score has long been used as an important reference for predicting surgical mortality and morbidity. In a prospective study, ChatGPT-4 was evaluated in 2,851 patients and achieved a kappa score of 0.858, indicating almost perfect agreement with anaesthesiologists for ASA classification.^7^ In another study, ChatGPT was evaluated in 203 paediatric patients and achieved a kappa score of 0.72, indicating substantial agreement with anaesthesiologists.^8^ An algorithm developed using ML, based on data from 12,064 patients, achieved a 70.4% accuracy.^9^ These results suggest that AI systems can approximate anaesthesiologists’ decision-making in preoperative risk assessment consistently. ASA scoring can vary significantly among anaesthesiologists, which should be taken into account when evaluating the results of these studies.

### Depth of Anaesthesia

The optimal depth of anaesthesia is defined as a state in which unconsciousness, analgesia, and immobility are achieved without excessive sedation. Maintaining this equilibrium remains one of the most intricate challenges in contemporary anaesthesiology. Conventional practice continues to rely largely on physiological indicators, such as mean arterial pressure (MAP) and heart rate; however, the neuronal dynamics underlying consciousness extend far beyond the information these parameters can provide.

Electroencephalography (EEG) offers real-time insights into neuronal activity and enables the estimation of anaesthetic depth through characteristic waveform patterns. However, its clinical interpretation remains constrained by interindividual variability, the combined effects of anaesthetic agents, and the non-linear relationship between cortical activity and the level of consciousness.^10, 11^ In recent years, data-driven AI-based approaches have emerged to overcome these limitations in EEG analysis. Unlike conventional systems, such as the bispectral index, entropy, or patient state index, which rely on predefined mathematical models, ML algorithms can extract complex temporal and spectral features directly from EEG signals. Among these, convolutional neural networks (CNNs) and recurrent neural networks have demonstrated superior accuracy and speed in decoding the multidimensional structure of EEG data, extending well beyond the capabilities of traditional indices.^12, 13^ For instance, models employing deep residual shrinkage networks have achieved highly accurate predictions of anaesthetic depth by analysing 14 EEG-derived features.^12^ Similarly, CNN-based hybrid models have classified patients into four anaesthetic states, namely awake, light, general, and deep, with an accuracy of approximately 89%.^13^ These findings suggest that AI can detect subtle neural signatures in EEG activity before clinical or haemodynamic changes become apparent, potentially enabling earlier and more individualised control of anaesthetic depth.

AI-assisted EEG monitoring not only enhances accuracy but also improves interpretability under complex anaesthetic conditions involving multiple agents. Different anaesthetic drugs induce distinct EEG changes owing to their specific mechanisms of action.^14^ Because of this variability, single-index systems cannot maintain the same level of sensitivity across all situations. ML models trained on large and multidimensional datasets that incorporate these diverse patterns can adapt to both drug- and patient-specific characteristics.^15^ Consequently, anaesthetic titration can be guided on a more physiological basis, reducing the risk of intraoperative awareness or excessive suppression.

### Hypotension Prediction Index

Intraoperative hypotension is a silent yet devastating complication of anaesthesia. When MAP falls below 65 mmHg, particularly when the duration of this decline is prolonged, irreversible impairments in the perfusion of the heart, kidneys, and brain may occur, increasing the risk of postoperative organ dysfunction and mortality.^16^ However, in clinical practice, intraoperative hypotension is often recognised and treated only after it has developed. However, this reactive approach is often insufficient to prevent physiological injuries.

The hypotension prediction index (HPI) represents a pivotal step toward proactive haemodynamic management. Using an AI algorithm that analyses 23 features derived from the arterial pressure waveform, the system can estimate the likelihood of MAP dropping below 65 mmHg several minutes in advance.^17^ Early validation studies have demonstrated that the algorithm can predict hypotension 5, 10, and 15 min before the onset, with sensitivities and specificities exceeding 80%.^18^ This early warning capability provides clinicians with a critical window to initiate timely, targeted interventions such as fluid optimisation or vasopressor titration. Recent systematic reviews have shown that this technology may translate not only into physiological benefits but also into measurable clinical outcomes. A meta-analysis evaluating the use of HPI in noncardiac surgery reported that, compared with standard care, HPI-guided management significantly reduced the total hypotension burden (area under the hypotensive threshold, mean difference -60.28 mmHg·min), the incidence of hypotension (mean difference -4.50), and the cumulative duration of hypotension (mean difference -12.8 min).^19^ In another contemporary meta-analysis of 19 studies, HPI-guided therapy was associated with a significant reduction in intraoperative hypotension and related major complications (relative risk 0.79; 95% confidence interval 0.69-0.90).^20^ These findings suggest that HPI may shift anaesthetic management from a reactive practice to a proactive haemodynamic paradigm.

Nevertheless, the concept of “predictive haemodynamics” embodied by the HPI has been increasingly scrutinised. Analyses of large databases have indicated that although the algorithm demonstrates high technical accuracy, its clinical impact appears to be less pronounced. Recent prospective studies have reported that, despite strong discriminative performance [receiver operating characteristic-area under the curve (AUC) ≈ 0.9], the positive predictive value of HPI remains approximately 30%.^21, 22^ Furthermore, several investigations have shown that many of these predictions can be replicated by simpler models that rely solely on current MAP trends.^23^ Given that MAP is among the dominant features within the 23 parameters analysed by the algorithm, some authors have argued that HPI may primarily reflect the existing haemodynamic state rather than an independent predictive signal.^24^ As a result, it remains uncertain whether HPI provides a clinically meaningful advantage over conventional MAP-based monitoring.

Despite these promising features, the cost-effectiveness of HPI-guided monitoring remains a major practical barrier. The technology requires proprietary hardware-software integration and additional disposables, which substantially increase per-patient monitoring costs.

### Echocardiography

Echocardiography is the most dynamic reflection of cardiac function, particularly in the setting of cardiac anaesthesia. The clarity of this mirror depends largely on human factors (e.g., operator expertise and speed of interpretation) and on image quality. Under anaesthesia, where haemodynamic conditions can shift within seconds, rapid and accurate assessment of ventricular performance directly influences clinical outcomes. AI aims to redefine this complex equation by transforming echocardiography from a purely visual assessment tool into a quantitative, standardised, and predictive analytical platform.

In recent years, ML and DL algorithms have been developed to automatically identify cardiac chambers and compute left ventricular volumes and ejection fractions within seconds. The AutoLV software introduced by Knackstedt et al.^25^ analysed ejection fraction and strain in 255 patients across four centers in just eight seconds, demonstrating a strong correlation with manual measurements. Similarly, Asch et al.^26^ evaluated 279 ejection-fraction datasets and showed that DL-based analysis achieved an accuracy comparable to expert interpretation. The AutoVTI function (GE Healthcare, Chicago, IL), which enables real-time and continuous measurement of stroke volume through velocity-time integral analysis, has further facilitated the continuous monitoring of cardiac output trends using echocardiography.^27^

The impact of AI is not limited to the assessment of left ventricular function. Strain analysis evaluates ventricular deformation and provides insights beyond conventional ejection fraction measurements. ML-based algorithms have achieved high accuracy in identifying ventricular dysfunction in patients with heart failure with preserved ejection fraction.^28^ Liu et al.^29^ demonstrated a strong correlation between AI-assisted strain measurements and manually obtained fractional area change (FAC) in the evaluation of right ventricular function. However, correlations involving TAPSE were weak or nonsignificant when compared with FAC and global longitudinal strain.^29^ In mitral valve analysis, 3D echocardiography has significantly improved measurement accuracy and clinical decision support by leveraging its greater data capacity. Jeganathan et al.^30^ analysed intraoperative 3D transoesophageal echocardiographic data from four patients undergoing coronary artery bypass grafting, using the eSie Valve software (Siemens Healthcare, Mountain View, CA), which automatically calculated six geometric parameters of the mitral annulus and leaflets. In this study, full-volume mitral valve datasets were obtained over two to three cardiac cycles without R-wave synchronization, including the entire annulus and the coaptation region. The results demonstrated that the mitral valve could be analysed fully automatically and reproducibly throughout systole and diastole, with reduced dependence on user input. Similarly, the “AI in ultrasound” method enhanced diagnostic accuracy and consistency, even among novice users, by enabling semi-automated analysis of annular and leaflet structures to identify mitral valve prolapse.^31^ However, this system was designed solely for prolapse detection and remains limited in identifying other pathologies, such as clefts or chordal rupture.

Despite these advances, several important limitations remain in the integration of AI into echocardiography systems. Image quality is the most critical factor directly affecting algorithm performance, and the margin of error increases under conditions such as atrial fibrillation, obesity, and poor acoustic windows.

### Airway

The rapid advances in AI and imaging technologies have ushered in a new era in predicting difficult intubation and improving intubation safety. AI has demonstrated significant progress in predicting difficult intubation using models based on facial morphology analysis. Cuendet et al.^32^ developed a fully automated system that predicts difficult intubation by evaluating morphological features automatically extracted from facial photographs of 970 patients and applying a random forest algorithm; the system achieved an AUC of 77.9%. Similarly, Connor and Segal^33^ classified difficult intubation using a computerized analysis model combining facial ratios and the thyromental distance, achieving high accuracy (90% sensitivity, 85% specificity, AUC=0.899) and significantly outperforming classic clinical tests. These studies demonstrate that AI can be a powerful tool for predicting difficult airways from facial images. Although facial-image-based AI models demonstrate impressive predictive performance, their actual integration into routine preoperative workflow remains questionable. Obtaining standardized facial photographs, ensuring proper lighting and positioning, securing patient consent, and transferring images to an AI introduce additional steps that are often impractical in a busy preoperative clinic. Moreover, variability in camera quality and environmental conditions may undermine model accuracy in real-world settings.

Lakhani^34^ used DL models to detect the presence of an endotracheal tube (ETT) on radiographs (AUC, 0.99) and to determine tube position (AUC, 0.81). This study is among the first robust demonstrations of the high performance of X-ray-based AI for ETT detection. Han et al.^35^ successfully performed a preoperative assessment of a laryngectomized patient with a difficult airway by creating a 1:1 tracheal model from computed tomography (CT) images using a 3D printer. The 3D model clearly showed the anatomical changes in the airway, ensuring a safe anaesthesia plan for the patient. This study is one of the early and important examples demonstrating that CT data can be an effective tool for planning difficult airways using 3D printing.

According to a study by Zang et al.^36^, video-assisted devices shorten intubation time, increase first-attempt success rate, and improve safety, particularly in difficult airway management. AI has shown promising results in areas such as automatic anatomy identification in laryngoscopy and fibreoptic bronchoscopy videos, filtering unusable frames, vocal cord motion analysis, and tumour and vascular structure segmentation. However, the lack of standardisation of AI applications and the need for large, multicentre clinical studies have been identified as significant gaps in the literature. A retrospective study developed a machine-learning model to predict extubation failure after general anaesthesia in adult patients undergoing head, neck, and maxillofacial surgery.^37^ Of the 89,279 patients evaluated between 2015 and 2022, 77 experienced extubation failure; 186 successfully extubated patients were selected as controls matched by surgical procedure. Six different ML algorithms were tested using seven clinical variables identified by stepwise regression; the best performance was achieved with support vector machines and logistic regression (AUCs of 0.74 and 0.71, respectively). This study demonstrates that ML models can contribute to clinical decisions by enabling the early prediction of extubation failure after high-risk airway surgeries. AI algorithms have been trained to automatically identify pharyngeal and tracheal structures in videolaryngoscopy recordings. DL approaches have also been used to detect the laryngeal adductor reflex in laryngeal endoscopy videos, potentially enabling automated assessment of airway reflex integrity.^38^

Hypoxaemia is one of the most important complications of airway management, and clinicians’ predictions are often inadequate. Therefore, AI-based predictive models have attracted attention. For example, Lundberg et al.'s^39^ dynamic perioperative hypoxaemia prediction models demonstrated performance far beyond that of clinical assessment by analysing time-varying vital parameters, ventilator settings, and drug doses.

### Regional Anaesthesia

AI has also initiated a remarkable transformation in applications of regional anaesthesia, an area that is among the fastest-growing within anaesthesiology. It is widely accepted that one of the areas in which AI can contribute most effectively is regional anaesthesia, particularly through advances in imaging-based technologies.^40^ When the historical development of regional anaesthesia is examined, it is observed that neurostimulation techniques were integrated into clinical practice in the 1980s and ultrasound technology in the 2000s. Today, AI technologies have been integrated into this evolution and have begun to transform clinical training.^41^

AI-powered sonographic recognition systems can be integrated with conventional ultrasound devices, enabling AI-based identification of anatomical structures from image output (e.g., HDMI) regardless of image quality. Systems such as ScanNav^TM^ and Nerveblox have pioneered AI-based visual recognition technologies. A study using these systems showed that AI technology provided significant support in the real-time identification of anatomical structures by young anaesthesia physicians using ultrasonography.^42^ These findings highlight the increasing importance of AI-based visual recognition technologies in clinical education. In another study, data from 21 different practitioners were analysed; half of the practitioners were instructed to practice with the assistance of AI-supported systems, such as ScanNav^TM^. The results showed significant increases in the success of image acquisition, in the recognition of sonoanatomical structures, and in user satisfaction.^43^ Different versions of these systems are becoming increasingly common. It is predicted that NerveTrack, SmartNerve, and cNerve—systems with similar features—will become more widely adopted in clinical practice as software companies release and integrate them directly into ultrasound machines.^44^ Although these systems demonstrate substantial promise and have shown clear educational benefits, particularly for novice practitioners, their current role remains primarily supportive in training and skill acquisition. As highlighted in recent evaluations, AI-assisted recognition tools such as ScanNav^TM^ and Nerveblox are not yet capable of replacing expert sonographic judgment in complex or anatomically challenging cases. Instead, they function best as adjuncts that enhance learning and standardize training, rather than as autonomous clinical decision-makers.

The Q-VUM model developed by Wang et al.^45^ is a DL-based AI algorithm capable of recognising the quadratus lumborum (QL) muscle and surrounding anatomical structures in real time on ultrasound images. The model has shown potential for increasing the anatomical accuracy of QL blocks and reducing the risk of complications.^45^ As in many areas of anaesthesia, the use of AI-based models in clinical decision-making is increasingly widespread in regional anaesthesia. Studies, particularly those conducted using hypothetical patient scenarios, have demonstrated the potential of AI in guiding clinicians. For example, in a study in which hypothetical patient samples with 10 different anticoagulation profiles were presented to AI systems for neuraxial anaesthesia guidance, the authors observed that LLMs could support some basic clinical decisions, but their accuracy decreased significantly in complex cases.^46^ A recent pilot study evaluated the potential of LLMs as clinical decision support tools in regional anaesthesia practice. The ability of four different AI platforms to respond to simple and complex clinical scenarios was evaluated. Significant deficiencies regarding clinical reasoning, justification, and consistency were found in all models, and incorrect responses were particularly evident in complex cases. These findings indicate that current LLM-based systems are not yet ready to provide direct clinical decision support in regional anaesthesia practices, but they do represent a valuable preliminary step for future “e-consult” models to be developed.^47^ A recently published study investigated the agreement between clinicians and AI applications in selecting the anaesthesia method for patients undergoing orthopaedic surgery and reported that the Gemini application matched anaesthesiologists’ preferences 68.5% of the time. However, the study emphasized that it would be more appropriate to use these systems as decision support tools rather than replacing physicians, due to the AI’s inability to fully master clinical guidelines and its inadequate performance in specific patient groups.^48^

The Guidance for Reporting Artificial Intelligence Technology Evaluations for Ultrasound Scanning in Regional Anesthesia (GRAITE-USRA) guideline, published in 2025, is the first international reporting framework developed for the scientific evaluation of AI applications in ultrasound-guided regional anesthesia. This guideline, jointly approved by multiple international associations, aims to standardise elements such as study design, operator experience, ultrasound protocol, and data security through a 40-item checklist. The adoption of GRAITE-USRA will significantly contribute to the quality of academic publications and regulatory processes by increasing the comparability of the clinical validity of different AI-based systems.^49^ In parallel, the international Delphi consensus published by Fettiplace et al.^50^ emphasises transparency, ethical responsibility, and human oversight in the use of AI for scientific writing and publishing. Both guidelines provide a strong roadmap for integrating AI into regional anaesthesia in a manner that is methodologically sound and ethically aligned.

### Critical Care

AI has rapidly become one of the most dynamic frontiers in critical care medicine, with applications in prediction, diagnosis, monitoring, decision support, and operational optimisation. Since the first reports of AI-based intensive care analytics appeared in the late 2010s, its use has expanded from small proof-of-concept algorithms to comprehensive multimodal systems capable of integrating vast amounts of physiological, imaging, and electronic health record data. Its usefulness is paramount, considering that modern intensive care units (ICUs) generate enormous volumes of continuous, heterogeneous data (vital signs, laboratory values, waveform outputs, and clinical notes) that far exceed human cognitive capacity. AI offers a framework to transform these data into clinically actionable insights, thereby augmenting clinical decision-making and potentially improving patient outcomes.^51^

Predictive analytics is one of the most extensively studied domains. ML models have been developed to identify the early signs of deterioration, including sepsis, respiratory failure, and haemodynamic instability. For instance, DL frameworks trained on high-frequency ICU data have been shown to predict the onset of sepsis several hours before traditional diagnostic criteria would allow.^52^ Similarly, models integrating electronic health record data and bedside monitor streams have achieved impressive discrimination in predicting mortality and unplanned readmission in both adult and paediatric critical care.^53, 54^ These predictive systems exemplify AI’s potential to act as an early warning adjunct, allowing clinicians to intervene before physiological collapse occurs.

Risk classification and prediction of perioperative complications are central to ensuring surgical safety and informing surgical planning. Hospital records contain rich data, and AI has begun exploiting this potential: in a prospective study, ChatGPT correctly predicted postoperative ICU admission for 65.5% of 406 patients, but performed poorly for other outcomes such as ICU length of stay.^55^ In another study using 10 preoperative clinical scenarios, ChatGPT failed to meet minimum clinical standards for perioperative planning, underscoring that current LLMs should be viewed as adjuncts, rather than autonomous decision-makers, in preoperative risk assessment.

AI is also revolutionising clinical decision-making. Algorithms embedded within decision support systems can recommend ventilator settings, antibiotic regimens, and fluid strategies by continuously analysing the evolving patient data. Such systems do not replace the clinician but rather provide dynamic, data-driven suggestions that refine judgment and standardise care.^56^ In perioperative and neurocritical care, AI-enhanced monitoring has been used to titrate anaesthetic depth and cerebral perfusion more precisely; in trauma and postoperative management, AI models have guided resuscitation and analgesic dosing.^57^ These studies highlight AI’s role as an augmentative rather than an autonomous agent, expanding clinicians’ situational awareness in data-dense environments.

Another key area is multimodal monitoring and data integration. Intensive care involves the continuous collection of physiological signals, such as ECG, EEG, and arterial pressure waveforms. ML techniques, including convolutional and recurrent neural networks, can process complex time-series data to detect patterns invisible to human observers. For example, predictive algorithms have been used to identify early signs of acute respiratory distress syndrome and to forecast the need for mechanical ventilation.^58, 59^ In parallel, NLP is increasingly employed to mine unstructured clinical notes and nursing reports, allowing AI to detect subtle clinical shifts or errors that structured data may miss.^60^

Imaging analysis is another promising avenue. DL networks trained on radiographic and ultrasound data can automatically detect pneumothorax, effusion, or ventilator-associated changes, providing near-real-time diagnostic support in settings where expert interpretation may be delayed.^61^ Similarly, AI-augmented echocardiography and CT analysis have improved the recognition of cardiac dysfunction and cerebral oedema in critically ill patients.^62^ Beyond diagnostics, AI is being used to enhance procedural precision—for example, in needle placement and line insertion—through computer-vision-assisted feedback systems.^63^

Finally, several studies have examined AI’s role in resource optimisation and system management. Predictive algorithms can anticipate ICU bed occupancy, model staffing needs, and streamline triage by matching patient severity to appropriate care levels.^64^ In multicentre tele-ICU platforms, AI supports remote monitoring and triage prioritisation, improving response times in resource-constrained environments.^65^ These operational uses underscore how AI can optimise clinical outcomes and healthcare efficiency.

### Academic Literature

AI, particularly LLMs, has rapidly entered the academic ecosystem and is now being used in multiple stages of scholarly activity. These applications include idea generation, literature searching and synthesis, manuscript drafting, methodological assistance, and language refinement.^66, 67^ Despite the accelerating adoption of these tools and the policies introduced by major publishers and editorial organisations, a universally accepted guideline for the use of AI in academic writing has yet to be established. There is broad consensus in the scientific community that AI tools cannot meet the criteria for authorship. As noted by organisations such as the International Committee of Medical Journal Editors and Committee on Publication Ethics, AI lacks the capacity for accountability, cannot take responsibility for the integrity of the work, and is unable to approve the final version of a manuscript.

Therefore, transparency and human oversight are essential. Any use of AI within the research or writing process must be explicitly disclosed, specifying the tool, version, and precise purpose for which it was used. Importantly, AI should function only as a supportive instrument; the responsibility for scientific accuracy, ethical compliance, interpretation of findings, and data confidentiality remains with the authors. Authors must rigorously verify all AI-assisted outputs to ensure fidelity, methodological soundness, and adherence to ethical standards.^50^

### Anaesthesia Education

Specialty training in anaesthesia requires both theoretical knowledge and high-level technical skills. AI has frequently been used to improve and personalise education. A comprehensive review by Komasawa^68^ reported that AI can develop student-specific learning paths in anaesthesia education. The study emphasised that simulation-based applications can be personalised. This allows students to gain technical skills as well as awareness of patient safety and decision-making processes. Cai et al.'s^69^ study showed that AI-supported image-recognition systems contribute significantly to regional anaesthesia education. In this study, a training platform was developed to automatically identify nerve structures in ultrasound images using a convolutional neural-network-based algorithm. A randomised simulation study showed that the incidence of paraesthesia during puncture and injection was significantly lower among anaesthesia assistants using this system than among those using the traditional method. Such AI-supported tools not only accelerate the learning process but also have the potential to reduce the risk of complications in real-world clinical settings. Sardesai et al.^70^presented an innovative approach using AI-supported virtual patient simulations, particularly for preoperative consultation and training in patient communication. The vast majority of participants found these systems user-friendly, accessible, and realistic, demonstrating that AI can be used not only to develop technical skills but also to develop communication skills. However, the study reported that some responses were incorrect or inadequate. This finding indicates that human oversight remains critical for enhancing the effectiveness of AI-based simulations as educational tools.

A review published by Yu et al.^71^ suggested that the integration of AI and virtual reality technologies could be used particularly for patient safety and crisis management training. Such systems enable the repeated practice of high-risk scenarios in a safe environment, thereby improving decision-making skills under stress. However, the authors emphasised that these technologies must be rigorously evaluated for their validity and reliability before being integrated into training programs. Jin et al.^72^ evaluated ChatGPT’s success in creating learning materials for anaesthesia assistants. Ninety-five prompts derived from Anaesthesia Knowledge keywords (focused information, broad response/compilation, lesson plan, “biased/incorrect” prompts, and reference prompts) were submitted to ChatGPT-3.5 and ChatGPT-4.0. The responses were scored by two experienced anaesthesiologists on a three-point scale for accuracy and, for long responses, completeness. Ultimately, 55% of the responses were deemed completely accurate by both evaluators, and accuracy and completeness for broad-scope prompts were mostly rated at the “book/expert” level. However, significant errors and fabricated (hallucinated) references were observed in prompts containing incorrect assumptions and in cases where literature references were requested. It was emphasised that some errors could cause harm in clinical practice. The authors suggest that while ChatGPT can be useful and generate consistent content on entry-level topics, it is not reliable on its own in medical education; its outputs need to be verified by expert review, and field-specific (anaesthesiology) knowledge bases and user training are important considerations.

An overview of the major benefits and opportunities of AI in anaesthesiology, along with its current challenges and limitations, is presented in Tables 1 and 2.

## Conclusion

AI is rapidly reshaping anaesthesiology, offering unprecedented opportunities to enhance perioperative safety, precision, and efficiency. AI-driven tools are increasingly demonstrating their value as augmentative decision-support systems in risk stratification, airway assessment, haemodynamic optimisation, echocardiography, regional anaesthesia, critical care, and medical education. However, significant challenges, including data quality, transparency, algorithmic bias, regulatory uncertainty, medico-legal accountability, and the risk of overreliance, highlight the need for cautious, evidence-based integration. AI will not replace anaesthesiologists; rather, it will redefine the profession toward a more cognitive, supervisory, and data-informed role. The safe and meaningful adoption of AI requires robust validation, interdisciplinary collaboration, clinician training, ethical governance, and transparent human oversight. As AI systems continue to evolve, future research must prioritise clinically relevant outcomes, multicentre evaluations, interpretability, and equitable access.

## Figures and Tables

**Table 1. Benefits and Opportunities of AI Integration in Anaesthesiology and Perioperative Medicine:** 

**Enhanced risk predictions**	AI enables large-scale data screening to improve risk prediction models, increasing diagnostic accuracy and early warning capabilities. These systems can estimate mortality, postoperative complications, ICU admission, and length of stay. Such predictions support informed consent, perioperative planning, and resource allocation.
**Prediction of specific complications**	Multimodal AI models synthesizing physiological signals, laboratory data, and imaging can identify early signs of cardiovascular instability, renal dysfunction, and ventilatory disorders before clinical deterioration. Future AI-driven advanced monitoring may integrate haemodynamic, respiratory, and neurophysiological parameters to deliver real-time recommendation-based support.
**Personalized risk assessments**	AI utilizes patient-specific variables (e.g., ASA class, airway features, comorbidities) to generate individualized risk estimates, facilitating personalized perioperative planning and enhancing risk-benefit discussions with patients.
**Precision and decision-making aid**	AI algorithms analyze EEG, ECG, and haemodynamic patterns to help clinicians titrate anaesthetic and vasoactive drugs more precisely. Closed-loop systems using AI-derived depth of anaesthesia and haemodynamic indices can autonomously adjust infusion pumps, minimizing variability, reducing adverse effects, and improving outcomes.
**Imaging and procedural guidance**	AI strengthens image interpretation in echocardiography and regional anaesthesia by assisting with nerve identification and pattern recognition. It improves image acquisition, reduces operator variability, and enables automated calculations (e.g., EF, SV, CO), enhancing speed, accuracy, and reproducibility.
**Workflow efficiency and administrative support**	AI reduces administrative workload by transcribing clinical conversations, drafting perioperative documentation, and generating discharge summaries. AI-driven predictions of surgical duration, theatre utilization, and patient flow improve operational efficiency. In ICUs, AI assists in forecasting length of stay, readmission risk, and resource needs.
**Education and communication**	AI supports patient engagement, preoperative education, psychological reassurance, and multilingual communication. In medical training,AI enhances self-directed learning and powers advanced simulation platforms, including virtual OR scenarios, to improve teamwork and crisis management skills.
**Acceleration of drug discovery**	AI accelerates anaesthetic drug development by enhancing large-scale data analytics, toxicity prediction, and clinical trial optimization. Tools such as AlphaFold help identify protein targets, deepening understanding of anaesthesia mechanisms and enabling faster, more efficient pharmacologic innovation.

AI, artificial intelligence; ICU, intensive care unit; ASA, American Society of Anesthesiologists; EEG, electroencephalography; ECG, electrocardiography; EF, ejection fraction; SV, stroke volume; CO, cardiac output; OR, operating room

**Table 2. Challenges and Limitations of AI Integration in Anaesthesiology and Perioperative Medicine:** 

**Technical and methodologic limitations**	AI models often function as opaque “black boxes,” preventing reverse engineering or clear interpretability. This reduces clinician trust and complicates accountability and medicolegal responsibility. These issues parallel proprietary EEG-based anaesthesia depth monitors, where simplified indices obscure underlying algorithmic processes.
**Data quality, volume, and generalizability**	AI performance is constrained by the quality and diversity of training data. Many studies rely on small or single-center datasets, limiting external validity. Models perform poorly in rare diseases or complex decisions and remain vulnerable to overfitting, requiring continuous monitoring and retraining.
**Clinical safety and judgement risks**	AI models may hallucinate, generate factually incorrect or inconsistent outputs, and produce erroneous references, necessitating clinician oversight, especially in high-risk fields like anaesthesia and critical care where tolerance for error is minimal.
**Overreliance and automation bias**	Dependence on AI may diminish clinical engagement, critical thinking, and interpretive judgement. Automation bias can lead clinicians to uncritically accept AI recommendations, increasing the risk of adverse events.
**Data safety and ethical concerns**	AI relies on sensitive patient data, raising concerns about consent, privacy, and data security. Cloud-based systems are vulnerable to cyber-attacks, and healthcare settings are high-risk targets. Proposed mitigations include strong encryption, federated learning, and blockchain-based audit trails.
**Ethical, regulatory, and professional challenges**	Regulatory pathways (e.g., MDR, FDA) require extensive validation and can impose high costs. Hallucination-related errors may create malpractice liability. Clear protocols for responsibility within AI-assisted or closed-loop systems are needed. Ethical issues also include patient data use and the validity of informed consent when AI tools provide unreliable information.
**Education and compliance**	Many anaesthesiologists lack formal training in AI principles, data governance, algorithmic auditing, or troubleshooting. Without structured AI curricula in residency and continuing education, clinical adoption will face resistance and misuse risks.
**Implementation and infrastructure costs**	AI deployment requires substantial investment in hardware, software, cybersecurity, cloud storage, governance structures, and dedicated IT personnel. These financial and operational burdens challenge implementation in resource limited healthcare systems.

EEG, electroencephalography; MDR, medical device regulation; FDA, food and drug administration; AI, artificial intelligence; IT, information technology
